# Association of claw disorders with subclinical intramammary infections in Egyptian dairy cows

**DOI:** 10.14202/vetworld.2017.358-362

**Published:** 2017-03-28

**Authors:** Walid Refaai, Medhat Gad, Yasser Mahmmod

**Affiliations:** 1 Department of Surgery, Anesthesiology, and Radiology, Faculty of Veterinary Medicine, Zagazig University, 44511 Zagazig, Sharkia Province, Egypt; 2 Directorate of Veterinary Medicine, Sharkia Branch, Zagazig, Sharkia Province, Egypt; 3 Department of Veterinary and Animal Sciences, Faculty of Health and Medical Sciences, University of Copenhagen, DK-1870 Frederiksberg C, Denmark; 4 Department of Animal Medicine, Faculty of Veterinary Medicine, Zagazig University, 44511 Zagazig, Sharkia Province, Egypt

**Keywords:** claw affections, dairy cows, digital dermatitis, subclinical mastitis

## Abstract

**Aim:**

Bovine mastitis and lameness are the most common production diseases affecting dairy farms worldwide resulting in huge economic impact and impaired animal welfare. The objective of this field study was to investigate the association of infectious and non-infectious claw disorders with the occurrence of subclinical intramammary infections (IMIs) diagnosed by California mastitis test (CMT) in dairy cows under Egyptian conditions.

**Materials and Methods:**

A total of 43 dairy cows were included in this field study. Subclinical IMI was diagnosed by CMT on all lactating quarters of cows. A cow was considered to have subclinical IMI if it had at least one subclinically infected quarter (≥3). Cows were inspected carefully for claw disorders that recorded based on type and site. Locomotion and body condition scores were also recorded for each cow in addition to the limb affected. The association between the CMT and other explanatory variables was tested by Fisher’s exact test.

**Results:**

The prevalence of infectious and non-infectious claw disorders was 81.4% (35/43) and 32.6% (14/43), respectively. Digital dermatitis (DD) and heel horn erosion were the most prevalent infectious type with 79% (34/43) and 58% (25/43), respectively, while wall fissure was the most identified non-infectious one 11.6% (5/43). The prevalence of claw disorders in hind limbs was 88.4% (38/43) and 11.6% (5/43) in the forelimbs. Infectious claw disorders were significantly associated with the subclinical IMI diagnosed by CMT (p<0.05). Non-infectious claw affections, locomotion score, body condition score, and the affected limb had no association with the occurrence of subclinical IMI.

**Conclusion:**

DD is the highest prevalent claw disorder observed in dairy cows in Egypt. The hind limbs are more susceptible to claw disorders than the forelimbs. Infectious type of claw disorders is significantly associated with subclinical IMI diagnosed by CMT in dairy cows under Egyptian conditions indicating that the infectious types of claw affections may influence the udder health.

## Introduction

Subclinical intramammary infections (IMIs) are considered the most common disease limiting the production of dairy farms worldwide. It is associated with a large economic impact and impaired animal welfare [1]. The economic importance associated with subclinical IMIs stems from reduction of milk quality and quantity [2]. Moreover, the absence of clinical symptoms for IMI and lack of quality control system in some developing countries hinders early detection and accurate diagnosis and subsequent, implementation the prevention and control measures against the disease.

The California mastitis test (CMT) has been accepted as a practical and simple cow-side test for screening of subclinical IMI in dairy cows under field conditions based on the DNA content of the milk [3]. CMT is a rapid and inexpensive test to indirectly determine the somatic cell count (SCC) in milk [4]. Identifying the risk factors associated with subclinical IMI is crucial in designing the appropriate prevention and control measures. Numerous risk factors for IMI such as parity, milk yield, SCC, stage of lactation, teat lesions or calloused teats have been identified [3,5,6]. However, there is no readily-available information or literature on the association between claw disorders and occurrence of subclinical IMI in dairy herds.

Lameness in cattle is a major cause of economic losses in dairy production [7]. Claw disorders have been found to be the cause of more than 90% of all lameness cases observed in dairy cattle [8]. Claw disorders are either infectious or non-infectious [9]. Digital dermatitis (DD) is considered an important cause of lameness in dairy farms, with economic losses due to weight loss, dropped milk yield, impaired fertility, and costs of treatment [8,9]. The association between bovine claw specific lesions and milk yield in Egyptian dairy farms has been reported previously in many research papers [10-12]. Green *et al*. [13] found that the total mean estimated reduction in milk yield per 305-day lactation was approximately 360 kg indicating that clinical lameness has a significant impact on milk production. Moreover, it was found that claw diseases were significantly associated with clinical mastitis [14]. There is no available literature that described the association between claw disorders as a common cause of lameness and subclinical IMI in dairy cows. We hypothesize that claw disorders would predispose the occurrence of subclinical IMI and this may depend on the type of claw disorders. The objective of this field study was to investigate the association of infectious and non-infectious claw disorders with the occurrence of subclinical IMI diagnosed by CMT in dairy cows under Egyptian conditions.

## Materials and Methods

### Ethical approval

All applicable international, national, and/or institutional guidelines for the care and use of animals were followed. All procedures performed in the study involving animals were in accordance with the ethical standards of the institution or practice at which the study was conducted.

### Study population and cow enrolment

The study was conducted on one large dairy herd (n=300 Holstein–Friesian cows) with conventional milking system in Sharkia province, Egypt, between March 2015 and July 2015. The dairy herd has frequent occurrence of lameness despite the hygiene program for claw health. The farm was visited once weekly and cows recruited for the study were among those that were lame.

Before each visit, the dairy farmers isolated cows that had abnormal gait after exit from milking parlor so that preliminary examination as well as scoring their body and locomotion can be done. Thorough examination of the cow’s feet was performed in a claw-trimming box for the detection of claw disorders. From the total number of examined cows, only 43 were identified to have claw disorders and were registered in the data sheet. Cows were housed outdoor on an earthen floor and were fed on concentrate and silage. All cows in the study were kept under the same feeding regime, environment, and management practices for the whole study period.

### Data collection

#### Subclinical mastitis

Subclinical mastitis was diagnosed by performing the CMT on all lactating quarters of each cow under the study. The CMT technique was performed as described by Schalm and Noorlander [15] for each milk sample collected at the farm. Briefly, the first stream of milk was discarded and then a few streams of milk were collected in the corresponding paddle wells. The paddle was tilted to remove excess milk, and an equal amount of commercial reagent (DeLaval mastitis test CMT, DeLaval operations, Poland) was added to each cup. Gentle circular motion was applied in a horizontal plane for 15 s to mix the milk with the reagent.

The result was scored on a five-point scale with score one as completely negative, score three as clearly positive and score five as the maximum based on the viscosity of the gel formed by mixing the reagent with the milk [16]. A cow was considered to have subclinical mastitis if it had at least one subclinically infected quarter.

### Cow scoring and claw disorders

On the visiting day, these separated cows were thoroughly examined for claw disorders. Briefly, cows were carefully inspected by physical clinical exploration to describe site, type, and extent of the lesion. All four feet were examined and findings recorded. Lesions were classified based on their potential etiology, as either infectious or non-infectious causes of lameness. Lesions classified as infectious causes of lameness were DD, interdigital necrobacillosis, interdigital dermatitis (IDD), and heel horn erosion (HHE). Claw disorders classified as non-infectious causes of lameness were sole hemorrhage, sole ulcer (SU), wall fissure (WF), white line disease (WLD), Subclinical laminitis (LAM), double sole, interdigital hyperplasia (IH), Toe necrosis (TN), and Bulb ulcer (BU)

The body condition was scored using five-point scale (1=emaciated to 5=obese) with increments of 0.25 [17]. The dataset of each cow including ear tag identification number, position of affected limb/s, and description of claw lesions (site and type) which were associated with the lameness were recorded.

Locomotion scoring for each cow was performed according to Sprecher *et al*. [18] on a five score scale based on posture and gait of the cow, where score 1 representing normal locomotion and scores 2, 3, 4 and 5 representing lame animals. A cow was considered to have lameness if it had a locomotion score > one. Full description of the locomotion scoring system and assessment criteria are presented in Table-1.

**Table-1:** Description of locomotion scoring system applied in our study based on criteria used to assign a lameness score and clinical description to dairy cows developed by Sprecher et al. (1997).

Lameness score	Clinical description	Assessment criteria
1	Normal	The cow stands and walks with a level-back posture. Her gait is normal
2	Mildly lame	The cow stands with a level-back posture but develops an arched-back posture while walking. Her gait remains normal
3	Moderately lame	An arched-back posture is evident both while standing and walking. Her gait is affected and is best described as short striding with one or more limbs
4	Lame	An arched-back posture is always evident and gait is best described as one deliberate step at a time. The cow favors one or more limbs/feet
5	Severely lame	The cow additionally demonstrates an inability or extreme reluctance to bear weight on one or more of her limbs/feet

### Statistical analysis

Before undertaking statistical analysis, data were screened for unlikely or missing values. Subsequently, an initial exploratory analysis including summary statistics for the different variables was performed. The association between the subclinical IMI based on CMT and explanatory variables including body condition score, position of the lame leg, locomotion score, and infectious and non-infectious claw disorders was investigated.

For the analyses, CMT was dichotomized (<3, ≥3) where a cow with at least one quarter showing CMT score ≥3 was considered as having subclinical IMI. Locomotion score was dichotomized (<2, ≥2) where cow with locomotion score ≥2 was considered as lame. Lameness was categorized as front-leg or hind-leg lameness, according to the position of the lame leg.

Body condition score was dichotomized (<3, ≥3). A cow was considered as positive for infectious claw condition if she had at least any one among DD, IDD, or HHE. A cow was also considered to have non-infectious claw condition if she had at least one among conditions such as SU, WF, WLD, LAM, IH, TN, and BU

Given the small sample size (n=43), the association between the CMT and other explanatory variables was tested by Fisher’s exact test. Significance for statistical analysis was defined as p<0.05, and the analysis was performed using R v3.0.3 (R Core Team. R: A language and environment for statistical computing. <http://www.R-project.org/> [R Foundation for Statistical Computing, Vienna, Austria, 2015]).

## Results

A total of 43 cows had been enrolled at the end of the study period and had complete data observations. DD and HHE were the most prevalent infectious claw disorders with 79% (34/43) and 58% (25/43), respectively, while WF was the most identified non-infectious claw disorder 11.6% (5/43). Description of potential risk factors is presented in Table-2. The prevalence of claw disorders in the hind limbs was 88.4% (38/43) and 11.6% (5/43) in the forelimbs. The frequency and distribution of the infectious and non-infectious claw disorders are shown in Table-3. The prevalence of infectious and non-infectious disorders was 81.4% (35/43) and 32.6% (14/43), respectively. Association of potential risk factors with subclinical IMI diagnosed by CMT is shown in Table-4. Infectious claw disorders were significantly associated with subclinical IMI diagnosed by CMT (p<0.05).

**Table-2:** Descriptive statistics of variables of interest that were considered in the analysis for association of claw disorders with subclinical IMIs diagnosed by CMT in Egyptian dairy cows.

Variable	Level	Number of observations
Lame leg position	1=Right front	2
	2=Right hind	22
	3=Left front	3
	4=Left hind	16
Locomotion score	0=Negative (<2)	10
	1=Positive (≥2)	33
CMT score	0=Negative (<3)	19
	1=Positive (≥3)	24
Body condition score	0=Negative (<3)	12
	1=Positive (≥3)	31

CMT=California mastitis test, IMIs=Intramammary infections

**Table-3:** Infectious and non-infectious claw disorders and the corresponding CMT results as an indicator for subclinical IMIs in Egyptian dairy cows.

Claw disorders	Number of cases	CMT (%)	

		0=Negative	1=Positive
Infectious			
DD	34	17	17
IDD	4	3	1
HHE	25	14	11
Non-infectious			
SU	2	0	2
WF	5	2	3
WLD	2	1	1
LAM	2	2	0
IH	2	1	1
TN	1	0	1
BU	1	1	0

DD=Digital dermatitis, IDD=Interdigital dermatitis, HHE=Heel-horn erosion, SU=Sole ulcer, WF=Wall fissure, WLD=White line disease, LAM=Subclinical laminitis, IH=Interdigital hyperplasia, TN=Toe necrosis, BU=Bulb ulcer, CMT=California mastitis test, IMIs=Intramammary infections

**Table-4:** Association between potential risk factors and subclinical IMIs diagnosed by CMT in Egyptian dairy cows.

Variable	Level	Number of observation (%)	CMT (%)		p value

			0=Negative	1=Positive	
Lame leg position	1=Front	4	0	4	0.09
	2=Hind	39	19	20	
Body condition score	0: <3	12	4	8	0.29
	1: ≥3	31	15	16	
Locomotion score	0=No	10	4	6	0.53
	1=Yes	33	15	18	
Infectious claw disorders	0=Absent	8	1	7	0.05
	1=Present	35	18	17	
Non-infectious claw disorders	0=Absent	29	13	16	0.58
	1=Present	14	6	8	

CMT=California mastitis test, IMIs=Intramammary infections

## Discussion

Identifying the risk factors for subclinical IMI is essential for the implementation of effective control measures. Dairy cow lameness is a major problem for the dairy industry, causing reduced animal welfare and economic loss [19]. On the other hand, mastitis is one of the most frequent and costly disease of lactating dairy cows worldwide [20]. Therefore, early detection of mastitis and lameness reduces economic losses and increases the cure rate of infected animals [21]. The objective of this study was to investigate the association of infectious and non-infectious claw disorders on the occurrence of subclinical IMI diagnosed by CMT in dairy cows under Egyptian conditions. In this study, infectious claw disorders including DD, HHE, and IDD are significantly associated with the occurrence of subclinical IMI diagnosed by CMT in dairy cows and may increase the odds/probability of IMI. Our finding is consistent with Hagiya *et al*. [22] who found positive genetic correlations between SCC with mastitis and claw disorders incidence suggesting that reduce SCC in the early stages of lactation would decrease the incidence of both mastitis and claw disorders. A similar finding was reported by Sato *et al*. [14] who found that clinical mastitis was associated to claw problems. The same conclusion was reported by Sogstad *et al*. [23] who reported that claw and hock lesions are associated with poorer reproductive performance and some production diseases such as mastitis. Clinical findings showed that non-infectious claw disorders have no significant association with subclinical IMI. This comes in agreement with Hultgren *et al*. [24] who could not find a significant association between SU and some reproductive disorders including clinical mastitis and high milk somatic cell counts. The possible, plausible explanation could be that non-infectious claw disorders are stem from non-infectious causes such as mechanical, chemical, or physical reasons. On the other hand, infectious claw disorders such as DD are infectious in nature and caused by infectious pathogens such as *Fusobacterium necrophorum, Bacteroides* and *Treponemes* species. Such pathogens exaggerate/induce the cow immune system more than the non-infectious claw disorders. Therefore, the immune system in case of infectious claw disorders may suppress the general immune status and that will subsequently; provide a favorable condition for the udder pathogens to cause subclinical IMI. The association between subclinical IMI and infectious claw diseases particularly DD make some researchers to define the DD as “mastitis of the foot.” Furthermore, other predisposing factors related to the animal, environment, and management were important in the development of lameness in general and infectious claw diseases on particular. Hence, controlling such factors may diminish the incidence of subclinical IMI.

No significant association was found between locomotion score, the position of lamed leg, and IMI. This may be explained by the lack of power and number of observations due to the small sample size in this study. It was expected that higher locomotion score (severe degree of lameness) is associated with declining the general health status and immune system; therefore, the cow may be more liable/prone for other infection including IMI. In addition, it was found that the behind limbs were more affected with claw disorders than forelimbs. This is compatible with previous studies [8,25]. It was expected that claw lesions of the behind limbs would be responsible for higher incidence of mastitis as it is closer to the mammary tissue in contrast to the claw lesions of the forelimbs. Body condition score was not significantly associated with the occurrence of IMI in dairy cows. This was not surprising findings because there is no plausible biological reason for that hypothesized association. This finding was in accordance with Berry *et al*. [26] who found no significant relation between body condition score and clinical mastitis. The authors added that association of body condition score with udder health lacked the biological significance.

It is important to emphasize that the small number of samples (n=43) used for testing our hypothesis and pursuing the current research was a major limitation of this study, which was a constraint for employing further analytical methods. Therefore, we recommend that future studies should consider a larger sample size and consider other potential factors such as hygienic score and wounds/lesions on the tarsus.

## Conclusion

DD is the highest prevalent claw disorder observed in the selected study populations. The hind limbs are more susceptible to claw disorders than the forelimbs. The infectious claw disorders are significantly associated with subclinical IMI diagnosed by CMT in dairy cows under Egyptian conditions indicating that the infectious types of claw affections may influence the udder health.

## Authors’ Contributions

WR, MG and YM Conceived and designed the study, collected the data and executed the study. YM Analysed the data. WR and YM Interpreted the data and wrote the manuscript WR, MG and YM drafted and revised the manuscript. All authors have read and approved the final manuscript.
